# COGNITIVE DISPERSION IS ASSOCIATED WITH PROTECTIVE COGNITIVE AND SOCIODEMOGRAPHIC FACTORS IN MIDUS

**DOI:** 10.1093/geroni/igad104.3233

**Published:** 2023-12-21

**Authors:** Laura Klepacz, Jeremy Hamm, Eric Cerino

**Affiliations:** North Dakota State University, Fargo, North Dakota, United States; North Dakota State University, Fargo, North Dakota, United States; Northern Arizona University, Flagstaff, Arizona, United States

## Abstract

Research examining the relationship between an individual’s levels of variability across a range of cognitive domains (dispersion) suggests that higher dispersion may be an early indicator of future cognitive impairment. However, this literature is still in its infancy and more research is needed to systematically examine the correlates of dispersion in large-scale, national samples that include individuals across the adult lifespan. Our study thus examined the relationship between cognitive dispersion across 6 tasks, mean cognitive performance, and sociodemographic characteristics in a national sample of adults from the Midlife in the United States (MIDUS) study (n=4,198; age 55±12.45). Dispersion was calculated by generating an intraindividual standard deviation across 6 unique cognitive tasks spanning several neuropsychological domains. In contrast to past research, bivariate correlations indicated that higher levels of cognitive dispersion were associated with higher levels of mean performance across cognitive tasks (r=0.28), higher education (r=0.08), higher income (r=0.04), and younger age (r=-0.07) (all ps<.05). The positive association between cognitive dispersion and mean performance was strongest for the episodic memory and working memory tasks. These results were consistent in both wave 2 and 3 of MIDUS and replicated in regression analyses that also revealed an increase in dispersion across waves 2 and 3 of MIDUS was associated with an increase in mean performance across the two waves (b=0.05, p<.001). Results of our study point to the possibility that, in certain unimpaired adult lifespan populations, dispersion may be associated with protective cognitive and sociodemographic factors linked to healthy cognitive aging.

